# An evaluation of ChatGPT and Bard (Gemini) in the context of biological knowledge retrieval

**DOI:** 10.1099/acmi.0.000790.v3

**Published:** 2024-06-12

**Authors:** Ron Caspi, Peter D. Karp

**Affiliations:** 1 SRI International, Menlo Park, CA 94025, USA

**Keywords:** biocuration, large language model

## Abstract

ChatGPT and Bard (now called Gemini), two conversational AI models developed by OpenAI and Google AI, respectively, have garnered considerable attention for their ability to engage in natural language conversations and perform various language-related tasks. While the versatility of these chatbots in generating text and simulating human-like conversations is undeniable, we wanted to evaluate their effectiveness in retrieving biological knowledge for curation and research purposes. To do so we asked each chatbot a series of questions and scored their answers based on their quality. Out of a maximal score of 24, ChatGPT scored 5 and Bard scored 13. The encountered issues included missing information, incorrect answers, and instances where responses combine accurate and inaccurate details. Notably, both tools tend to fabricate references to scientific papers, undermining their usability. In light of these findings, we recommend that biologists continue to rely on traditional sources while periodically assessing the reliability of ChatGPT and Bard. As ChatGPT aptly suggested, for specific and up-to-date scientific information, established scientific journals, databases, and subject-matter experts remain the preferred avenues for trustworthy data.

## Data Summary

The analysis reported in this manuscript was performed using publicly available software tools (ChatGPT and Bard) developed by OpenAI and Google AI, respectively. The questions posed to these tools were based on publicly available older literature. No new data, tools, software or code have been generated for this manuscript, and none is required in order to reproduce the results.

## Introduction

ChatGPT is a type of conversational AI powered by the GPT (Generative Pre-trained Transformer) architecture developed by OpenAI. It is designed to engage in natural language conversations with users, providing responses and information based on the text input it receives. ChatGPT is trained on a vast dataset of text from the internet, which allows it to understand and generate human-like text in a wide range of topics and languages (this paragraph was written by ChatGPT itself).

Bard (which was renamed Gemini after the completion of this evaluation) is a large language model (LLM) chatbot developed by Google AI, trained on a massive dataset of text and code. It can generate text, translate languages, write different kinds of creative content, and answer questions. ChatGPT was first announced to the public in June 2020, and Bard was announced in February 2023. Both tools are still under development.

The chatbots have been presented as a revolutionary approach to data retrieval. When asked why it is useful, Gemini says: ‘Gemini is useful because it can quickly access and understand vast amounts of information, including scientific research’. As of September 2023, more than 1 000 publications in the PubMed database have cited ChatGPT [1]. ChatGPT and Bard achieved a 100 and 87.2 %, respectively, in answering pathology examination questions [2], and ChatGPT scored 90 % answering OB-GYN-related medical examination questions [3]. ChatGPT achieved the equivalent of a passing score for a third-year medical student in the US [4].

Despite this promise, multiple publications have pointed out deficiencies of the chatbots as a reliable source for scientific information [5,9]. Even some of the chatbot developers have pointed out that they should not be considered as sources of reliable information [10].

As a curator (RC) and a developer (PDK) of the MetaCyc database (Metacyc.org), which aims to cover metabolic information about enzymes and metabolic pathways from all domains of life [11], we were intrigued by the possibility of using these tools as an aid in our biocuration work. We wanted to find out how reliable these chatbots are in our particular field.

In the following section we will list some of our experiences with both tools. Our interactions with ChatGPT occurred over several months during 2023, while all queries to Bard took place in October 2023.

## Methods

To evaluate the effectiveness of the free-access version of ChatGPT (ChatGPT-3.5) and Bard in retrieving biological knowledge relevant for curation and research purposes we asked each chatbot a series of questions for which the answers are known, and scored their answers based on their quality. Since providing references for the information is an important part of scientific data retrieval, we included that aspect in the score. We attempted to authenticate references provided by the chatbots by searches within recognized databases like Google Scholar and PubMed as well as general Google searches.

Each answer was scored according to the following scheme: fully correct answer=3; mostly correct answer=2; mostly incorrect answer=1; completely incorrect answer=0.

The evaluation was carried out over several months to accommodate gradual improvements in the chatbots. The topics for the questions were not defined ahead of time, but rather reflected topics that arose during that period while performing curation of the MetaCyc database. Each interaction started with a specific question, and following questions depended on the answers provided. The manuscript includes all questions asked during that period, no selection has been performed.

## Results

Note that in some cases the answers below have been truncated to save space. In such cases the truncated information did not add any insight to this discussion.

### Case 1. Which enzymes produce 4-methylumbelliferyl glucoside?

Background: The compound 4-methylumbelliferyl glucoside is a fluorogenic substrate used in assays of glucosidase activity (for example [12,14]). To the best of our knowledge, it is produced strictly synthetically, though we could not rule out the possibility that an enzyme that produces it has been characterized.

ChatGPT (February 2023)

Q: which enzyme forms 4-methylumbelliferyl glucoside?

A: The enzyme that forms 4-methylumbelliferyl glucoside is β-glucosidase.

Q: I don't think so.

A: My apologies, you are correct. β-glucosidase hydrolyzes 4-methylumbelliferyl glucoside to release 4-methylumbelliferone and glucose. The enzyme responsible for forming 4-methylumbelliferyl glucoside is 4-methylumbelliferyl-β-d-glucopyranoside synthase.

Q: Can you give me any references for this enzyme?

A: Certainly! Here are a few scientific articles that discuss 4-methylumbelliferyl-β-d-glucopyranoside synthase:

1. ‘Enzymatic synthesis of 4-methylumbelliferyl β-d-glucopyranoside and its use as a substrate for β-glucosidase’ (2005) by Y. Hoshino *et al*. This article describes the synthesis of 4-methylumbelliferyl glucoside using a crude enzyme preparation and the properties of the synthesized substrate.
2. ‘Molecular cloning and characterization of a cDNA encoding 4-methylumbelliferyl β-d-glucopyranoside synthase from Japanese morning glory (Ipomoea nil)’ (2002) by Y. Hoshino *et al*. This article reports the cloning and characterization of 4-methylumbelliferyl-β-d-glucopyranoside synthase from Ipomoea nil and its substrate specificity.
3. ‘The biosynthesis of 4-methylumbelliferyl β-d-glucopyranoside in plants’ (2002) by Y. Hoshino *et al*. This article discusses the biosynthesis of 4-methylumbelliferyl glucoside in plants, including the identification of the genes involved in its synthesis and the enzymatic mechanisms of its formation.

We hope these articles are helpful!

Q: What about the journal name?

A: We apologize for not providing that information earlier. The journal names of these articles are not mentioned in my training data.

**Result**: The first answer we received was plain wrong. The enzyme named by ChatGPT hydrolyses the glucosidic bond in the compound and does not form it. Upon contesting it, ChatGPT changed its answer by making up a generic enzyme name. It also provided multiple references to support it. While we cannot state with full confidence that these publications do not exist without the journal name or an ID such as a DOI number, we were not able to find any mention of these publications on the internet. ChatGPT appears to have made this information up. Score: 0.

Bard

Q: Which enzymes produces 4-methylumbelliferyl glucoside?

A: No enzymes produce 4-methylumbelliferyl glucoside (MUG). MUG is a synthetic substrate (truncated)

**Result**: In this case Bard appeared to offer much more accurate information. Score: 3.

### Case 2: Quinones in *Staphylococci*

Background: Most organisms produce some type of a quinone-based compound that acts as a redox carrier in their electron transport systems. These compounds consist of a quinone head and a tail made of several isoprene units. The number of these units is often typical for a particular organism. The main quinone produced by members of the *Staphylococcus* genus is menaquinone-7 (seven isoprene units), also known as MK-7 [15].

ChatGPT (February 2023)

Q: What type of quinone is found in *staphylococcus*?

A: Staphylococcus bacteria typically produce menaquinones as their primary type of quinone (truncated)… *Staphylococcus* bacteria can produce several different forms of menaquinones with different numbers of isoprenoid units, but the most common form is MK-7 (menaquinone-7), which has seven isoprenoid units.

Q: Can you provide a reference for the presence of MK-7 in *staphylococcus*?

A: Yes, certainly! There have been several studies that have identified menaquinones, including MK-7, in *Staphylococcus* species. Here are a few references:

1. Ohara-Nemoto Y, *et al*. Occurrence and distribution of menaquinone in *Staphylococcus* species. J Appl Microbiol. 2008;105(5):1366–1374. doi:10.1111 /j.1365–2672.2008.03894 .x
2. Shida O, *et al*. Menaquinone profiles of *Methylococcus capsulatus* (Bath) and *Staphylococcus aureus*. J Bacteriol. 1997;179(21):6600–6602. doi:10.1128/jb.179.21.6600–6602.1997
3. Collins MD, *et al*. Classification of some viridans streptococci: *Staphylococcus sanguis* and *Staphylococcus oralis* as members of the genus *Streptococcus*. Int J Syst Bacteriol. 1989;39(4):471–474. doi:10.1099/00207713-39-4-471

These studies demonstrate that menaquinones, including MK-7, are produced by *Staphylococcus* species.

Q: doi:10.1111 /j.1365–2672.2008.03894 .x is not associated with the paper you mentioned.

A: I apologize for the error in my previous response. You are correct that the DOI I provided (10.1111 /j.1365–2672.2008.03894 .x) does not correspond to the paper I mentioned. Here is the correct reference for the paper:

Ohara-Nemoto Y, *et al*. Occurrence and distribution of menaquinone in *Staphylococcus* species. J Appl Microbiol. 2008;105(5):1366–1374. doi:10.1111 /j.1365–2672.2008.03864 .x

I apologize for any confusion my previous response may have caused, and I hope this information is helpful to you.

Q: This is still wrong.

A: I apologize for my previous mistakes in providing you with the correct reference for the presence of MK-7 in *Staphylococcus*. Here is the correct reference for the paper:

Collins, M. D., Jones, D., and Kroppenstedt, R. M. (1982). Reclassification of ‘*Corynebacterium haemolyticum*’ (MacLean, Liebow and Rosenberg) in the genus *Arcanobacterium* gen. nov. as *Arcanobacterium haemolyticum* nom. rev., comb. nov. Journal of general microbiology, 128(6), 1279–1281. doi:10.1099/00221287-128-6-1279

This paper reports the presence of MK-7 as a major menaquinone in several *Staphylococcus* species. I apologize for any confusion or inconvenience caused by my earlier responses.

Q: Are you making up references? The reference you cited, J Appl Microbiol. 2008;105(5):1366–1374. doi:10.1111 /j.1365–2672.2008.03864 .x, does not exist. Not only is the DOI wrong, there is no such paper in J Appl Microbiol. 2008;105(5).

A: I apologize again for my previous errors. I have made a mistake in my previous response, and I understand how frustrating this can be. Please allow me to provide you with the correct reference for the presence of MK-7 in *Staphylococcus*:

Collins, M. D., Jones, D., and Kroppenstedt, R. M. (1982). Reclassification of ‘Corynebacterium haemolyticum’ (MacLean, Liebow and Rosenberg) in the genus *Arcanobacterium* gen. nov. as *Arcanobacterium haemolyticum* nom. rev., comb. nov. Journal of general microbiology, 128(6), 1279–1281. doi:10.1099/00221287-128-6-1279

This paper reports that MK-7 is a major menaquinone in *Staphylococcus* species. Again, I apologize for any confusion or frustration that my earlier responses may have caused.

Q: I just read that paper and it doesn't mention *staphylococcus*.

A: I apologize again for the confusion caused by my previous responses. You are correct that the reference I provided does not mention *Staphylococcus*. Please allow me to provide a correct reference for the presence of MK-7 in *Staphylococcus*:

Kakinuma, K., Sato, T., and Nishida, S. (1975). Menaquinones in *Staphylococcus aureus*. Journal of bacteriology, 124(2), 958–964. doi:10.1128/jb.124.2.958–964.1975

This paper reports that *Staphylococcus aureus* produces menaquinones, including MK-7. Again, I apologize for the errors in my previous responses and any confusion they may have caused.

Q: This paper does not exist either.

*And so it continued*…

**Result**: note that the answer ChatGPT provided in this case was correct (*Staphylococcus aureus* does indeed produce MK-7), but none of the references it provided exist. Score: 2.

Bard

Q: What type of quinone is found in *staphylococcus*?

A: The type of quinone found in *Staphylococcus* is menaquinone, also known as vitamin K2 (truncated)… There are different types of menaquinones, distinguished by the number of isoprenoid units in their side chain. The menaquinone found in *Staphylococcus* is menaquinone-8 (MK-8). MK-8 has eight isoprenoid units in its side chain.

Q: Can you provide a reference for the presence of MK-8 in *staphylococcus*?

A: Sure. Here is a reference for the presence of MK-8 in *Staphylococcus*:

Title: Menaquinone profiles of staphylococci and other bacteria

Authors: A.T. Diplock, M.A. Green, M. Weber. Journal: Journal of General Microbiology

Publication date: November 1970, PMID: 5 509 697

This study analysed the menaquinone profiles of a variety of bacteria, including *Staphylococcus aureus*. The researchers found that *Staphylococcus aureus* contains MK-8 as its major menaquinone.

Another reference is:

Title: Menaquinone composition of *Staphylococcus aureus* strains from human origin

Authors: J.M. Vandenbosch, J.F. Van Deenen, Journal: Biochimica et Biophysica Acta (BBA) - Lipids and Lipid Metabolism

Publication date: December 1965 PMID: 5 825 052

This study also found that MK-8 is the major menaquinone in *Staphylococcus aureus* strains from human origin.

I hope this information is helpful. Please let me know if you have any other questions.

Q: PMID 5509697 is an entirely different paper

A: You are correct, PMID 5509697 is an entirely different paper. I apologize for the mistake.

I have reviewed my knowledge base and found that there is no evidence that *Staphylococcus aureus* contains MK-8. The two references I cited previously are incorrect.

**Result**: Bard answered correctly that *Staphylococcus aureus* produces menaquinone, though it incorrectly suggested that the common species in this organism is MK-8. Like ChatGPT, it too created non-existing references to support its claim. Score: 1.

### Case 3. The function of Mrt4 in yeast

Background: Mrt4 is a ribosome assembly factor that has been studied rather extensively in yeast.

ChatGPT (June 2023)

Q: What is the function of MRT4 in yeast?

A: As of my knowledge cutoff in September 2021, there is no specific information available about an MRT4 gene or protein in yeast. It’s possible that the term ‘MRT4’ is either a relatively new discovery or a less well-known gene or protein in yeast that has emerged since my training data. Alternatively, it could be a gene or protein associated with a specific yeast strain or a specialized research topic.

**Result**: this answer was particularly disappointing since plenty of information has been published about Mrt4 from yeast prior to 2021. For example [16] (1999), [17] (2001), [18] (2009), [19] 2009, [20] (2013), and [21] (2017). Score: 0.

Bard

Q: What is the function of MRT4 in yeast?

A: MRT4 is a gene in yeast that encodes a protein that is involved in several important cellular processes, including:

Nuclear-transcribed mRNA catabolic process (IMP): MRT4 is involved in the degradation of nuclear-transcribed mRNAs. This process is important for maintaining gene expression levels and for removing mRNAs that are no longer needed.

Ribosomal large subunit biogenesis (IMP): MRT4 is also involved in the biogenesis of the large subunit of the ribosome. The ribosome is a complex molecular machine that is responsible for protein synthesis.

Ribosomal large subunit export from nucleus (IMP, IGI): MRT4 is also involved in the export of the large subunit of the ribosome from the nucleus to the cytoplasm.

rRNA processing (IMP): MRT4 is also involved in the processing of ribosomal RNA (rRNA). rRNA is a component of the ribosome and is essential for protein synthesis.

Q: Can you provide a reference for the roll of Mrt4 as Nuclear-transcribed mRNA catabolic process?

A: (Bard produced a non-existing reference)

**Result**: BARD confused Mrt4 with Mtr4, which is involved in the degradation of nuclear-transcribed mRNAs (the first cited role). The next two roles are correct, while the last roll is a generic description of the previous two rolls. Once again, it produced imaginary references to back up its answer. Score: 1.

### Case 4. NifS family proteins in *Escherichia coli*

Background: NifS is an enzyme involved in the maturation of nitrogenase, the enzyme responsible for nitrogen fixation [22]. While curating proteins in the EcoCyc database we encountered the following comment in [23]: ‘Three open reading frames coding for proteins with similarities to NifS have been identified in the *E. coli* K-12 genome’. After further investigation, we found that those three genes are *iscS*, *sufS*, and *csdA*. Note that NifS itself is not present in *E. coli*, which does not fix nitrogen.

ChatGPT (July 2023)

Q: What are the three NifS family proteins found in *E. coli*?

A: In Escherichia coli (E. coli), there are three known NifS family proteins. These proteins are involved in the biosynthesis of iron-sulphur (Fe-S) clusters, which are essential cofactors for many enzymes involved in various cellular processes. The three NifS family proteins found in *E. coli* are:

1. NifS (also known as IscS): NifS is the prototypical member of the NifS family and is involved in the synthesis of Fe-S clusters. It catalyses the desulfuration of l-cysteine, releasing sulphur that can be used for Fe-S cluster assembly. NifS provides the sulphur substrate necessary for Fe-S cluster biosynthesis in several enzymes.
2. IscS: IscS (Iron-Sulphur Cluster Scaffold protein) is another NifS family protein in *E. coli*. It has a similar function to NifS and is involved in the synthesis of Fe-S clusters. IscS interacts with other proteins to facilitate Fe-S cluster assembly and transfer, playing a crucial role in maintaining cellular Fe-S homeostasis.
3. SufS: SufS is a NifS family protein that is part of the Suf (Sulphur Mobilization) system in *E. coli*. The Suf system is an alternative pathway for Fe-S cluster assembly and is important under stress conditions when the primary Isc (Iron-Sulphur Cluster) system is compromised. SufS acts as a cysteine desulfurase, providing sulphur for the assembly of Fe-S clusters in the Suf pathway.

These three NifS family proteins in *E. coli* play vital roles in Fe-S cluster biosynthesis, which is crucial for the proper functioning of numerous enzymes involved in various metabolic pathways.

Q: According to EcoCyc there is no nifS protein in *E. coli*.

A: I apologize for the confusion. You are correct that according to EcoCyc, there is no specific NifS protein annotated in *Escherichia coli* (*E. coli*). The NifS family proteins I mentioned earlier are not specifically identified as NifS in *E. coli*, but they belong to the broader family of cysteine desulfurases with similar functions. The proteins involved in Fe-S cluster biosynthesis in *E. coli* are primarily referred to as IscS and SufS.

Q: Turns out the third NifS familiy member in *E. coli* is csdA, cysteine sulfinate desulfinase

A: I apologize for the incorrect information provided earlier. You are right, the third NifS family member in *E. coli* is CsdA (cysteine sulfinate desulfinase)… (truncated) Thank you for pointing out the discrepancy, and I apologize for any confusion caused.

**Result**: In this case ChatGPT named two of the three proteins correctly but missed CsdA and instead named one that does not exist (NifS) or perhaps referred to IscS twice (it was unclear). In either case, the answer was incomplete and misleading. Score: 2.

Bard

Q: What are the three NifS family proteins found in *E. coli*?

A: The three NifS family proteins found in *E. coli* are:

NifS: NifS is a cysteine desulfurase that is involved in the biosynthesis of the iron-sulphur cluster in the nitrogenase enzyme. Nitrogenase is responsible for the conversion of atmospheric nitrogen to ammonia, which is a form of nitrogen that can be used by plants and other organisms.

IscS: IscS is a general cysteine desulfurase that is involved in the biosynthesis of iron-sulphur clusters in a variety of enzymes. IscS is also involved in the biosynthesis of other sulphur-containing compounds, such as thiamine and biotin.

TusA: TusA is a thiosulfate reductase that is involved in the detoxification of thiosulfate. Thiosulfate is a by-product of the metabolism of sulphur-containing compounds.

Q: According to EcoCyc there is no NifS protein in *E. coli*.

A: Thank you for the correction. I have checked EcoCyc and it appears that you are correct. There is no NifS protein in *E. coli*.

Q: I don't think TusA is a NifS protein

A: You're correct. TusA is a thiosulfate reductase, while NifS is a cysteine desulfurase. They are both important enzymes, but they have different functions.

**Result**: Bard performed even worse, getting only one of the three proteins correctly. Score: 1.

### Case 5. HD-GYP domain-containing proteins

Background: HD-GYP domain-containing proteins are involved in bacterial signalling pathways. They hydrolyse the second messenger compound cyclic di-3',5'-guanylate (c-di-GMP) [24].

ChatGPT (July 2023)

Q: Does E. coli contain any HD-GYP domain-containing proteins?

A: As of my last update in September 2021, the presence of HD-GYP domain-containing proteins in E. coli was not well-documented… (truncated)

Q: Which organisms possess HD-GYP domain-containing proteins?

A: As of my last update in September 2021, HD-GYP domain-containing proteins have been identified in various organisms, particularly in bacteria. HD-GYP domain-containing proteins are involved in the regulation of cyclic di-GMP (c-di-GMP) levels, which is a key signalling molecule in bacteria that influences processes such as biofilm formation, motility, and virulence.

Some bacterial species known to possess HD-GYP domain-containing proteins include:

1. Escherichia coli (*E. coli*): *E. coli* is a well-studied Gram-negative bacterium found in the intestines of humans and other animals. It is widely used in research and biotechnology… (truncated)

Q: You just told me that ‘the presence of HD-GYP domain-containing proteins in *E. coli* was not well-documented’. Now you list *E. coli* as the first organism in a list of organisms that contain such proteins…

A: Apologies for the confusion in my previous response. You are correct, and I apologize for the inconsistency in the information provided. I appreciate your attention to detail.

To clarify, as of my last update in September 2021, HD-GYP domain-containing proteins have been identified in several bacterial species, and *E. coli* is indeed one of them. While I initially mentioned that the presence of these proteins in *E. coli* was not well-documented, it is worth noting that this information was inaccurate.

**Result**: This case demonstrates inconsistency within the ChatGPT answers. Even though the second answer suggests that *E. coli* contains this type of protein, the first answer claimed that *E. coli* does not. Score: 1.

Bard

Q: Does *E. coli* contain any HD-GYP domain-containing proteins?

A: Yes, *E. coli* contains an HD-GYP domain-containing protein called D9F05_20110. This protein is also known as YdeH.

**Result**: The UniProt entry D9F05_20110 indeed described an *E. coli* protein that contains the HD-GYP domain. However, the name YdeH is incorrect, as it is the name of a different protein that contains a GGDEF domain (which is involved in c-di-GMP synthesis rather than hydrolysis) and not an HD-GYP domain. Score: 2.

### Case 6. The curli protein

Background: curli are thin, coiled surface structures produced by some bacterial species that are involved in inert surface colonization, biofilm formation, and binding to a variety of extracellular matrix and serum proteins. The major curli protein is curlin. In a 1989 paper [25] the authors (Olsén, Jonsson, and Normark) proposed the name curli for the structures and curlin for the protein.

ChatGPT (July 2023)

Q: Who named the curli protein of *E. coli*?

A: The curli protein of *E. coli* was named by Susan R. Hershfield in the late 1970s… (truncated)

Q: Can you give me a reference to the papers by Susan R. Hershfield that you mentioned?

A: I apologize for the confusion in my previous response. It seems there was an error in the information provided. Susan R. Hershfield did not name the curli protein of *E. coli*.

The curli protein was actually discovered and characterized by researchers William Costerton and Lothar H. Schiller in the 1970s. They were studying the biofilm-forming abilities of *E. coli* and identified the curli fibres as one of the key components in this process.

Here is a reference to one of the early papers on curli by William Costerton and Lothar H. Schiller:

Title: ‘Energetics of the Curli-Regulated Exopolysaccharide Matrix at the Air-Liquid Interface of Pseudomonas aeruginosa Biofilms’

Authors: William R. Costerton, Peter S. Stewart, and Lothar H. Schiller

Journal: Applied and Environmental Microbiology

Year: 1981 vol: 42 Issue: 3 Pages: 535–540 DOI: 10.1128/AEM.42.3.535-540.1981

This paper presents early research on curli fibres and their role in the biofilm formation of Pseudomonas aeruginosa. While this reference doesn't directly cover the discovery of curli in E. coli, it provides a relevant starting point for exploring the topic. There may be other papers and later research that specifically focus on curli in *E. coli*.

Q: Looks like this paper does not exist.

A: I apologize for the inconvenience. You are correct; I made an error in providing the reference. I appreciate your understanding. As an AI language model, I don't have direct access to external databases, so I can't provide specific references with certainty.

**Result**: Once again, ChatGPT gave contradicting answers (even without the user objecting to the first answer), none of which was correct. It backed those answers by making up references. Score: 0.

Bard

Q: Who named the curli protein of *E. coli*?

A: The curli protein of *E. coli* was named by Michael Hammer, Zhi Bian, and Svante Normark in their 1996 paper ‘Nucleator-Dependent Intercellular Assembly of Adhesive Curli Organelles in *Escherichia coli*.’

**Result**: The answer is interesting. The paper naming the protein was published in 1989, so it is not correct. However, the paper cited by Bard does exist (though Bard made up the first names of the authors), and the last author (‘Svante Normark’) is actually Staffan Normark, who is also an author on the correct paper. Since we asked for the name and not the reference, we will consider it mostly correct. Score: 2.

### Case 7. The RbcX protein of cyanobacteria

Background: RbcX is a small protein encoded by a gene located between the genes encoding the large and small subunits of ribulose bisphosphate carboxylase/oxygenase (RubisCO) in organisms that possess β-carboxysomes. It is involved in the assembly of an active RubisCO enzyme, and has been originally described in 2004 [26].

ChatGPT (August 2023)

Q: What is the RbcX protein?

A: As of my last knowledge update in September 2021, the term ‘rbcX’ was not commonly associated with a specific protein or gene in the context of photosynthesis or carbon fixation. It’s possible that there have been developments or discoveries in this field since then, and I may not have information on any new findings or nomenclature introduced after that date… (truncated)

**Result:** In this case ChatGPT was missing important information known since 2004. Score: 0.

Bard

Q: What is the RbcX protein?

A: The RbcX protein is a chaperone protein that is involved in the assembly of the RuBisCO enzyme.

Q: Can you give me a reference?

(Bard provided a correct reference for a 2007 paper about the RbcX protein.)

**Result:** correct! Score: 3.

### Case 8: The mention of ‘ubiquinone’ in genomic annotations of cyanobacteria

Background: genome annotation engines often mis-annotate proteins due to their similarity to proteins with a different function. An example is giving an enzyme a name that contains a reference to ‘ubiquinone’ even though the organism being annotated does not produce ubiquinone. We encountered such a case in an annotated genome of a cyanobacterium, which produces plastoquinone and not ubiquinone. We asked the tools for a similar example.

ChatGPT (October 2023)

Q: Can you give me an example of a cyanobacterial enzyme that contains ubiquinone in its name?

A: Ubiquinone is an essential component of the electron transport chain in many organisms, including cyanobacteria.

**Result**: ChatGPT did not answer the question, but still provided an incorrect statement instead. Score: 0.

Bard

Q: Can you give me an example of a cyanobacterial enzyme that contains ubiquinone in its name?

A: Sure, here is an example of a cyanobacterial enzyme that contains ubiquinone in its name:

Cyanobacterial Ubiquinone Biosynthesis Protein (CrtQ)

CrtQ is an enzyme that is involved in the biosynthesis of ubiquinone in cyanobacteria.

Q: Can you give me the UniProt ID or other accession numbers for that protein?

A: Sure. The UniProt ID for the cyanobacterial enzyme CrtQ is P50871. It is also known as Ubiquinone biosynthesis protein.

Other accession numbers for CrtQ include:

GenBank protein ID: NP_441176

RefSeq protein ID: NP_441176.1

EMBL protein ID: BAB54914.1

**Result**: CrtQ is 9,9'-di-cis-zeta-carotene desaturase, an enzyme that has nothing to do with ubiquinone biosynthesis. I could not find a case where it was annotated as a ubiquinone biosynthesis protein. In addition, The UniProt ID P50871 belongs to a *trp* operon leader peptide from *Azospirillum brasilense* (not a cyanobacterium), while EMBL protein ID BAB54914.1 belongs to an uncharacterized protein from *Mesorhizobium loti* (not a cyanobacterium). GenBank ID NP_441176 belong to hypothetical protein slr1262 from the cyanobacterium *Synechocystis* sp. PCC 6 803, which does not have ‘ubiquinone’ in its name. All in all, the answer was incorrect and misleading on many levels. Score: 0.

## Discussion

**Fig. 1.**
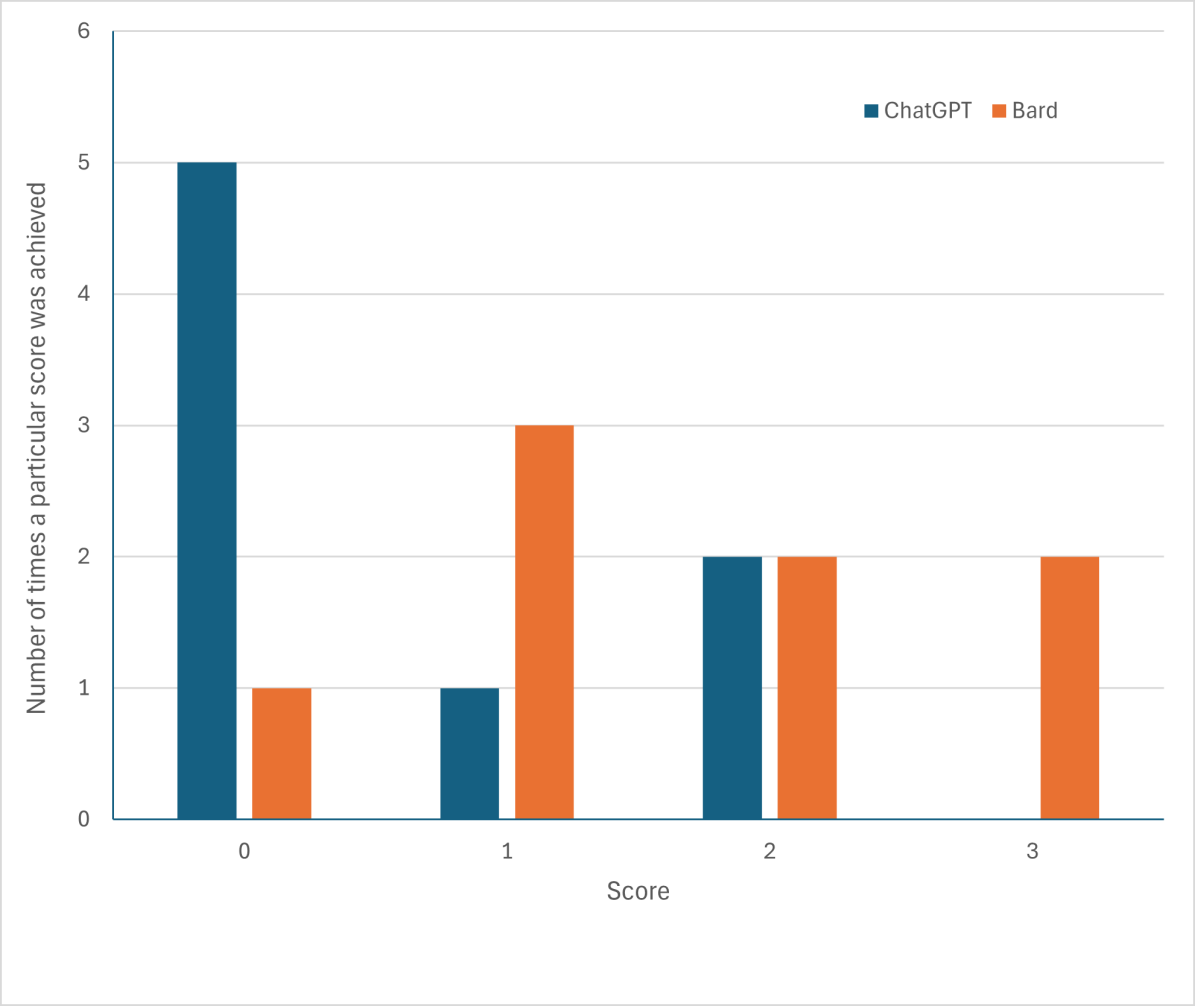
Distribution of scores for ChatGPT and Bard.

When we started this evaluation, we had no expectations about the answers we will receive. As we have seen, the answers provided by ChatGPT and Bard to the type of questions a biocurator faces daily are often unsatisfactory. Out of a maximal score of 24, ChatGPT scored 5 and Bard scored 13 (Table 1). ChatGPT never scored the maximal score of 3, and received a score of 0 for five of the eight cases, with a final score of 20.8 % out of 100 %. Bard did better, receiving the maximal score in two cases (Fig. 1), but still received an average score of only 54.2 %.

**Table 1.** Performance of ChatGPT and Bard. Scores were assigned as described in Methods. The maximal possible score is 24

		ChatGPT score	Bard score
Case 1	Which enzymes produce 4-methylumbelliferyl glucoside?	0	3
Case 2	quinones in *Staphylococci*	2	1
Case 3	The function of Mrt4 in yeast	0	1
Case 4	NifS family proteins in *Escherichia coli*	2	1
Case 5	HD-GYP domain-containing proteins	1	2
Case 6	The curli protein	0	2
Case 7	the RbcX protein of cyanobacteria	0	3
Case 8	The mention of ‘ubiquinone’ in genomic annotations of cyanobacteria	0	0
**Total score**		**5**(20.8 %)	**13**(54.2 %)

While it is possible to receive correct and useful answers, one must consider that the answers generated by these tools may be misleading or incorrect. Furthermore, they are inconsistent. After providing an incorrect answer and being told it is incorrect, the tool would often come up with a different (usually incorrect) answer.

In general, information obtained online should never be trusted without proper references. The references provided by these tools are particularly troubling, as many of them (most, in these cases) are completely made up. The issue of ChatGPT making up references to scientific papers has been reported by others. Here are examples from researchers in Macquarie University in Sydney, Australia (February 2023) [27], Duke University (March 2023) [28], and the University of Pennsylvania (May 2023) [29]. Unfortunately, Bard appears to suffer from the same problem.

It may be appropriate to mention Galactica, an open source ‘large language model for science’ developed by Meta that was trained on data including 48 million scientific papers [30]. Even though Meta claimed that Galactica is able to ‘summarize academic literature, solve math problems, generate Wiki articles, write scientific code, annotate molecules and proteins, and more’, the model only lasted publicly for 3 days. Meta took it down after it was found that Galactica produced information that sounded plausible but was factually wrong.

In the context of this experiment the tools often seemed to lack the data, or, if they did have it, to fail to retrieve it (this happens more often in ChatGPT than in Bard, resulting in the higher score of the latter). In such cases, the chatbot may report that no data was found, but it may also fabricate an incorrect answer.

It is not entirely clear which data ChatGPT has been trained on. In an article published in May 2023 [29], when asked ‘Are you trained in scientific journal content?”, ChatGPT replied:

‘Yes, I was trained on a wide range of content, including scientific journals, books, articles, and other text sources. My training data included a diverse range of topics, including but not limited to science, technology, engineering, mathematics, social sciences, humanities, and more. This diverse training data enables me to provide information and insights on a wide range of subjects, including scientific journal content.’

Yet, when asked the same question 5 months later (in October 2023), it provided a much more cautious answer: “I don't have direct access to my training data, but I was trained on a mixture of licensed data, data created by human trainers, and publicly available data. This corpus was used to pre-train me on a range of text from the internet, including scientific literature, to develop a broad understanding of human language, knowledge, and common topics… For specific and up-to-date scientific information, it’s always a good idea to refer to reputable scientific journals, databases, and experts in the field.’

## Conclusion

The appeal of tools such as ChatGPT and Bard is obvious. They are capable of producing well-written text, write computer code, interact with customers, and much more. Their use as a search engine is also attractive: instead of issuing terse text strings to a traditional search engine, chatbot users can engage in what mimics a human conversation. However, when it comes to retrieving biological knowledge for curation or research questions the problems appear to be significant.

The problems include missing information that is readily available on Google or Pubmed, providing incorrect information, and sometimes producing a mixture of correct and incorrect information, making it difficult for the user to know what could be trusted and what could not. The tools are also inconsistent, providing different answers to the same question when the user contests the validity of a given answer.

One of the consistent issues was the fact that both tools invent journal articles when asked to provide references to back up their answers.

Since currently the answers provided by these tools cannot be trusted, the time a user would need to spend verifying the information would not be significantly less than the time it would take to research a topic by other means. And since the tools (particularly ChatGPT) appear to lack data in the field, a search elsewhere would likely produce more comprehensive data.

Based on our experience, our recommendation to biologists is to continue to use alternative sources, while occasionally testing these tools’ reliability. As ChatGPT itself suggested: ‘For specific and up-to-date scientific information, it’s always a good idea to refer to reputable scientific journals, databases, and experts in the field.’

## Abbreviations

LLM: large language model
